# A Glimpse of Streptococcal Toxic Shock Syndrome from Comparative Genomics of *S. suis* 2 Chinese Isolates

**DOI:** 10.1371/journal.pone.0000315

**Published:** 2007-03-21

**Authors:** Chen Chen, Jiaqi Tang, Wei Dong, Changjun Wang, Youjun Feng, Jing Wang, Feng Zheng, Xiuzhen Pan, Di Liu, Ming Li, Yajun Song, Xinxing Zhu, Haibo Sun, Tao Feng, Zhaobiao Guo, Aiping Ju, Junchao Ge, Yaqing Dong, Wen Sun, Yongqiang Jiang, Jun Wang, Jinghua Yan, Huanming Yang, Xiaoning Wang, George F. Gao, Ruifu Yang, Jian Wang, Jun Yu

**Affiliations:** 1 Beijing Genomics Institute, Chinese Academy of Sciences, Beijing, China; 2 Department of Epidemiology, Research Institute for Medicine of Nanjing Command, Nanjing, China; 3 Institute of Microbiology and Epidemiology, Academy of Military Medical Sciences, Beijing, China; 4 Center for Molecular Immunology, Institute of Microbiology, Chinese Academy of Science, Beijing, China; 5 James D. Watson Institute of Genome Sciences of Zhejiang University, Hangzhou, China; 6 School of Biosciences and Bioengineering, South China University of Technology, Guangzhou, China; 7 The Institute of Human Genetics, University of Aarhus, Aarhus, Denmark; 8 China-Japan Joint Laboratory of Molecular Immunology and Molecular Microbiology, Institute of Microbiology, Chinese Academy of Sciences, Beijing, China; 9 Graduate University, Chinese Academy of Sciences, Beijing, China; Duke University Medical Center, United States of America

## Abstract

**Background:**

*Streptococcus suis* serotype 2 (SS2) is an important zoonotic pathogen, causing more than 200 cases of severe human infection worldwide, with the hallmarks of meningitis, septicemia, arthritis, etc. Very recently, SS2 has been recognized as an etiological agent for streptococcal toxic shock syndrome (STSS), which was originally associated with *Streptococcus pyogenes* (GAS) in *Streptococci*. However, the molecular mechanisms underlying STSS are poorly understood.

**Methods and Findings:**

To elucidate the genetic determinants of STSS caused by SS2, whole genome sequencing of 3 different Chinese SS2 strains was undertaken. Comparative genomics accompanied by several lines of experiments, including experimental animal infection, PCR assay, and expression analysis, were utilized to further dissect a candidate pathogenicity island (PAI). Here we show, for the first time, a novel molecular insight into Chinese isolates of highly invasive SS2, which caused two large-scale human STSS outbreaks in China. A candidate PAI of ∼89 kb in length, which is designated 89K and specific for Chinese SS2 virulent isolates, was investigated at the genomic level. It shares the universal properties of PAIs such as distinct GC content, consistent with its pivotal role in STSS and high virulence.

**Conclusions:**

To our knowledge, this is the first PAI candidate from *S. suis* worldwide. Our finding thus sheds light on STSS triggered by SS2 at the genomic level, facilitates further understanding of its pathogenesis and points to directions of development on some effective strategies to combat highly pathogenic SS2 infections.

## Introduction

*Streptococcus suis* (*S. suis*) infection is notorious for causing serious zoonotic diseases manifesting as meningitis, septicaemia, arthritis, etc. in addition to its great economic impact on swine industries worldwide each year[1], [2]. Since its discovery in Denmark in 1968, SS2, the most prevalent serotype among the 35 known serotypes, has spread to nearly 20 countries and has caused more than 200 human infections[1], [3], [4]. Notably, two recent large-scale outbreaks of human STSS caused by SS2 in China in 1998 and in 2005 have posed public health concerns worldwide[3]–[5]. Infection is characterized by acute high-fever, vascular collapse, hypotension, shock, and multiple organ failure, as well as the short course of disease (acute death occurs within ∼2 hours of infection) and high mortality (97.4%)[3], [4].

In general, STSS refers to an extremely serious human infection that was initially believed to be caused by *S. aureus* [6], [7], but was later found to also be a sequel of GAS infections[8]–[10]. So far, superantigens have been demonstrated to show a correlation with the clinical manifestations of STSS[6], [7], [11]. Very recently, M protein, a highly conserved component of GAS cell wall, was also shown to be a virulence-associated factor (VAF) and to play a critical role in the molecular/pathological machinery of STSS[8], [12]. However, STSS due to infection with non-GAS streptococci, such as GCS, is poorly understood[13]. For instance, nothing is known at the molecular level about STSS caused by SS2[3]–[5].

Pathogenicity islands (PAIs), one kind of distinct genetic element, are considered as a subgroup of genomic islands (GI) which may be acquired by horizontal gene transfer (HGT)[14]–[18]. Currently, it is accepted that PAIs may contribute to virulence in a wide range of pathogenic bacteria such as *E. coli* [14], [19], *S. aureus* [6], [20], [21], *Salmonella* [22], [23], *Enterococcus faecalis* [24] and *S. pneumoniae* [25]. Fortunately, the availability of genomics, a robust tool in the research fields of bacterial genomic plasticity or pan-genome [26], [27], molecular variation or evolution[28]–[30], and pathogenesis or virulence mechanism[30]–[33] will make it possible to identify and characterize novel PAIs that may be linked to the molecular mechanisms of STSS caused by SS2.

In our continued endeavors to understand both STSS outbreaks in China (one in 1998, and the other in 2005), a novel PAI-like DNA segment of ∼89 kb in length was identified. Notably, it is only present in the Chinese virulent SS2 strains (98HAH12 and 05ZYH33) but not in P1/7, a European reference strain of highly virulent SS2. Moreover, it possesses nearly all the properties shared by known PAIs, with a mosaic architecture characteristic of PAI[15]–[18]. Subsequent experimental evidence further supported that it is specific to the Chinese strains of highly invasive SS2 that are responsible for STSS. To our knowledge, this is the first report documenting a PAI from *S. suis*, and our finding will provide a novel genome-wide insight into STTS caused by SS2, which may facilitate understanding of its pathogenesis and development of effective strategies to combat highly pathogenic SS2 infections in the near future.

## Methods

### Bacterial Strains and Growth

*S. suis* strains (40 in total) were cultivated for serotype confirmation and molecular techniques such as isolation of genomic DNA (Protocol S1)[3].

### Genome Sequencing and Comparative Genomics

Three *S. suis* genomes (98HAH12, 05ZYH33&05HAS68) were sequenced and assembled using the routine random shotgun method[33]. The assembled genome sequences (accession number CP000407, CP000408, and AARD00000000) were processed through the BGI (Beijing Genomics Institute) annotation pipeline which is a collection of software (Protocol S1). Subsequently, the above genomes available (note that 05HAS68 was only an unfinished draft genome) combined with that of P1/7 (available at the Sanger Center (www.sanger.ac.uk)) were employed to perform comparative genomics using BLASTN and BLASTX at the whole-genome level. Additionally, the genomic co-linearity of three genomes (98HAH12, 05ZYH33&P1/7) was generated[33]. Analysis of single-nucleotide polymorphisms (SNPs) was also carried out (Protocol S1).

### Search for PAI Candidate

To probe the possible existence of genomic islands (GIs) that may include PAIs in the Chinese SS2 strains, a combined search method was developed[17] (Protocol S1). By considering the acquired potential GIs together with the key clues from comparative genomics, we unexpectedly observed that an element designated 89K may be a putative PAI. We then identified and characterized the typical features of 89K which were shown to match the general criteria for known PAIs (Protocol S1).

### Determination of PAI Specificity

To test the strain-specificity of 89K, 3 sets of unique primers (Table S1) were designed to perform PCR-based examination on a total of 40 SS2 strains that included 30 Chinese isolates and 10 international strains. In particular, the Chinese strains consisted of 23 extremely virulent isolates from both SS2 outbreaks in China, 5 avirulent isolates, and 2 less virulent strains.

### Preliminary Expression Analysis of 89K, A Candidate PAI

To initially understand whether the expression of 89K is constitutive, RT-PCR was applied to monitor transcriptional levels of a two-component signal transduction system (TCS) in 89K. This TCS harbors a DNA-binding response regulator (05SSU0943) and a sensor histidine kinase (05SSU0944). Finally, the corresponding expression analysis was conducted by standard RT-PCR with 16S rRNA as control (Protocol S1).

### Experimental Animal Infection

To further differentiate the pathogenicity of the Chinese isolates of SS2, pig infection experiments were conducted in a bio-safety level 3 (BSL-3) facility[3]. SPF-pigs (4 per group, 2 for intravenous injection and 2 for intranasal inoculation) were challenged with strains 98HAH12, 05ZYH33 or S10. Avirulent strain 05HAS68 was used as negative control. The challenged piglets were monitored for clinical signs of disease every 3 hrs.

## Results

### General Genomic Features

**Figure 1:**
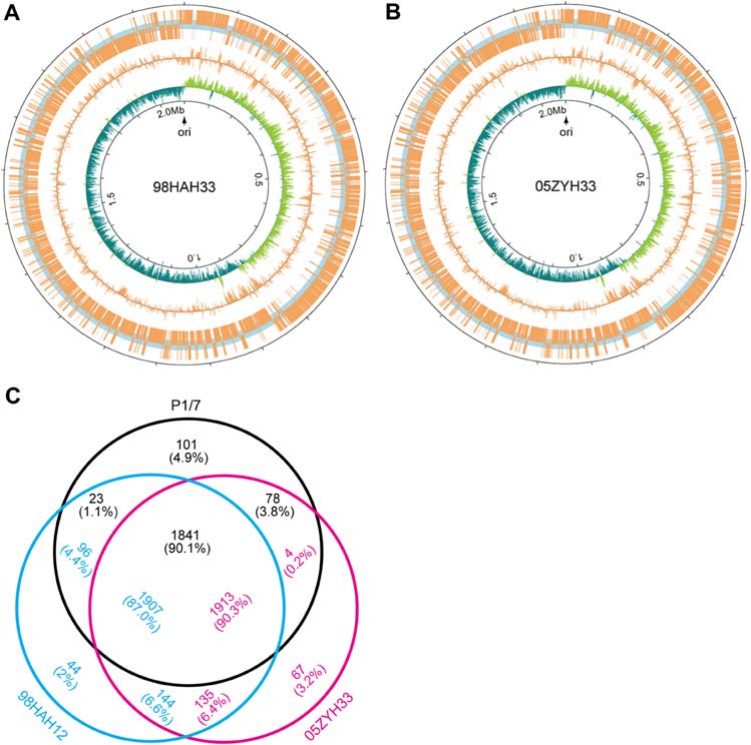
Circular diagrams and *in silico* comparison of two *S. suis* genomes. The two genome maps from 98HAH12 (A) and 05ZYH33 (B) were annotated with CGView (Stothard and Wishart 537-39). The replication origins of the genomes were determined using GC skews, dnaA boxes, and characteristic genes around the replication origins. The origins (indicated with vertical blue lines) are assigned as base pair 0 and then the sequences and their annotation are displayed clockwise. ORFs and their coordinates are displayed along both outside and at the center the concentric circles. In circle 1, the ORFs (in orange-yellow) outside (inside) represent the genes located on the positive (negative) strand. In circle 2, the GC contents of the genomes are shown. In circle 3, GC skew [(G−C)/(G+C)] is displayed (Green indicates values>0, and deep-blue indicates values<0). (C) The genes of 98HAH12, 05ZYH33 and P1/7 were compared using FASTA3. These numbers are displayed in the same color as that of each strain (98HAH12, 05ZYH33, and P1/7). Numbers in the intersections indicate genes shared by two or three strains. First, 44, 67, and 101 is the number of the unique genes in 98HAH12, 05ZYH33, and P1/7, respectively. Second, 1907 represents the number of the genes of 98HAH12, which are shared by both 05ZYH33 and P1/7; 1913 indicates the number of the genes of 05ZYH33, which can be found in both 98HAH12 and P1/7; 1841 means the number of the genes of P1/7, which are common in both 98HAH12 and 05ZYH33. Third, the numbers 144 and 135 separately indicate the genes of 98HAH12 and 05ZYH33 which are only shared with each other and not with P1/7; the numbers 4 and 78 represent the genes of 05ZYH33 and P1/7, respectively, which are only shared with each other but not with 98HAH12; the numbers 23 and 96 separately imply the genes of P1/7 and 98HAH12, which are only shared with each other, but not with 05ZYH33. Notably, the numbers in the intersections are slightly different partially due to gene duplications in some strains.

We carried out whole-genome sequencing for two Chinese virulent strains: 98HAH12 and 05ZYH33, which were isolated from fatal cases of STSS in 1998 and 2005, respectively. As a control, we also sequenced an avirulent strain 05HAS68 from a healthy pig. We observed that both virulent Chinese SS2 genomes (98HAH12&05ZYH33) comprise a single circular chromosome (Fig. 1A&Fig. 1B) with a GC content of 41.11% (Table S2). The genomes are 2,095,720 bp and 2,096,331 bp in length respectively, encoding 2191 and 2194 predicted open reading frames (ORFs) (Table S2). Their replication origins were predicted to be located at the intergenic regions of *dnaA* and a putative transcription regulator. Approximately 75% of the predicted ORFs could be assigned biological functions (Table 1&Table S2). In addition, a draft genome of an avirulent strain 05HAS68 was sequenced with about 8-fold coverage; it is 2,090,019 bp in length with an average GC percentage of 41.7%. The whole-genome sequence of P1/7, which contains 2,007,491 bp and shares the average GC content of 41.3%, was also retrieved from Sanger center (www.sanger.ac.uk) for further comparative analysis. The major features of the above genomes are listed in Table 1 as well as some of the statistics of genomics (Table S2).

**Table 1:** Genome-wide display of virulence associated factors/pathways in SS2

Function category	Strains		
	98HAH12	05ZYH33	P1/7
**Adhesin**			
Fibrinogen binding protein (*fbp*)	1	1	1
Muramidase-released protein (*mrp*)	1	1	1
Agglutinin receptor	1	1	–
Streptococcal hemagglutinin protein	1	–	–
**Proteinase**			
Collagenase and related proteases	1	1	1
Immunoglobulin A1 protease (*Zmp*C)	1	1	1
C5a peptidase	1	1	1
Hyaluronidase	2	2	2
Autolysin	1	1	1
Extracellular protein factor (*epf*)	1	1	1
**Hemolysin**			
Hemolysin III homolog	2	2	2
Suilysin (*sly*)	1	1	1
Putative hemolysin	2	2	2
Pneumolysin (*ply*)	1	1	1
**Dnase**			
Exonuclease V	2	2	2
Exonuclease VII	2	2	2
Mg-dependent DNase	1	1	1
**Antiphagocytosis**			
Cps2	30	22	24
Choline binding protein D (*cbpD*)	1	1	1
IgG-binding protein	1	1	1
**Signal pathway**			
Two-component system (TCS)	15	15	13
**Secretory system**			
Type II secretion system	13	11	13
Components from Type III secretion system	1	1	1
Components from Type IV secretion system	4	3	–

– **not found**

### Comparative Genomics

To gain insights into the possible genomic clues to high pathogenicity and STSS, the genomes were analyzed of two virulent strains (98HAH12&05ZYH33) causing STSS, a European virulent strain (P1/7) not causing STSS and one avirulent isolate (05HAS68). A high degree of conservation was noted in the genome organizations and gene contents. Additionally, these genomes presented strong strand-biased gene distribution, i.e. the genes prefer to reside on the leading strand (Fig. 1).

In addition to the obvious phylogenetic relationship, comparison of the three genomes (98HAH12, 05ZYH33&P1/7) also provided key information about pan-genomics of SS2—the core genome consisted of nearly 1,900 common genes, while the number of strain-specific genes varies from 44 to 101 (Fig. 1C). To assess the effect of SNPs on the intraspecies differentiation of 3 SS2 strains, we carried out SNP analysis. From the homologous genes from the genomes of 05ZYH33 and 98HAH12, we found 744 synonymous substitutions (Ks) and 1,971 nonsynonymous substitutions (Ka). The Ks value is 0.0021 and an average ratio of Ka/Ks is 0.776 (05ZYH33 vs. 98HAH12). Similarly, we detected 433 Ks and 1,194 Ka in both 98HAH12 and P1/7, resulting in a Ks value of 0.0012, and an average Ka/Ks ratio of 0.816 (05ZYH33 vs. 98HAH12). Both Ka/Ks ratios do not seem to be significantly different from each other. Therefore, the overall intensity of the selective pressure among the above three SS2 strains may be similar. Nevertheless, the exact contribution of point mutations can not be determined with respect to the physiological activities and pathogenicity.

Like other Gram positive pathogenic bacteria, several general pathogenicity-related pathways were also identified at the genomic level, besides those documented as virulence-associated factors (VAF)[1], [2], such as capsular antigen (CPS), suilysin, extracellular protein factor (EF), muraminidase-released protein (MRP), and glutamate dehydrogenase (GDH). In detail, firstly, there are 15 groups of TCSs in 98HAH12 and 05ZYH33, while there are only 13 groups of TCSs existing in European strain P1/7 (Table 1). Secondly, more than ten Type II secretion systems were found in the genomes of 98HAH12, 05ZYH33 and P1/7 (Table 1). Thirdly, 98HAH12 and 05ZYH33 separately harbor 4 and 3 components from Type IV secretion systems (T4SS) respectively, while P1/7 lacks them (Table 1). In fact, increasingly cumulative evidence has suggested that TCSs coupled with quorum sensing can regulate the expression of virulence genes[34]–[36], and different types of secretion systems contribute greatly to bacterial pathogenicity [37]–[41].

### Pathogenicity Island Candidate

**Figure 2:**
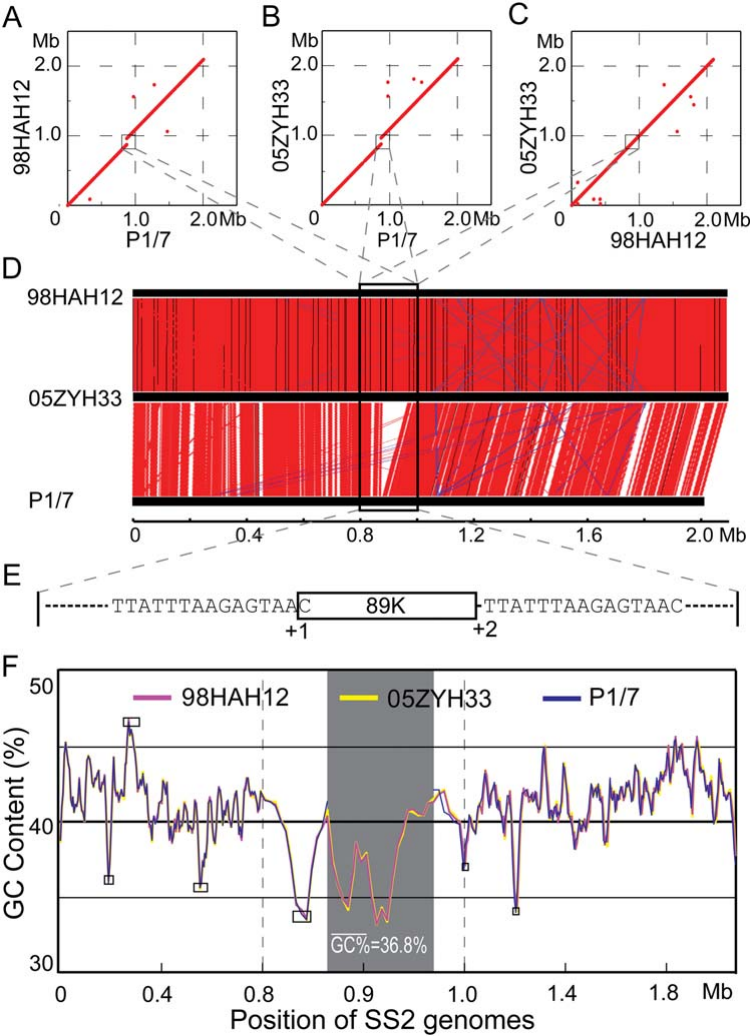
Identification of 89K, a putative PAI, through comparative genomics. (A) MUMer-based genomic display between 98HAH12 and P1/7. (B) MUMer-based genomic display between 05ZYH33 and P1/7. (C) MUMer-based genomic display between 98HAH12 and 05ZYH33. The hallmark in both (A) and (B) is a breakpoint of plots at the position of ∼0.9Mb which is highlighted in a square. (D) Co-linearity comparison of three *S. suis* genomes (98HAH12, 05ZYH33, and P1/7). The red lines represent similar DNA sequences (BLASTN search, *e*-value<10^−5^) between genomes. Blue lines stand for those regions with inverted sequences. Noticeably, a special region (designated 89K in a black rectangle) is present in both 98HAH12 and 05ZYH33, but absent in P1/7. (E) Mimic model of 89K in the vicinity of the genomes. The adjacent DNA sequences to 89K on both sides are directly presented. +1 means the first nucleotide acid (C) of 89K, and+2 indicates the second nucleotide acid (T) in the right arm of 89K. Obviously, 89K is highlighted in figures from (A) to (E), and linked each other by the dashed lines. (F) The GC contents of three *S. suis* genomes. The zigzags representing the GC percentage of *S. suis* strains 05ZYH33, 98HAH12&P1/7, are plotted in yellow, magenta and blue, respectively. The scale is amplified at genomic position from 0.8 to 1.0 Mb. The shadowed region represents the ∼89K segment in 05ZYH33 and 98HAH12. An artificial 89K gap is arranged in the P1/7 genome at the counterpart position. The boxes indicate the potential genomic islands in the *S. suis* genomes, excluding the ∼89K region.

**Figure 3:**
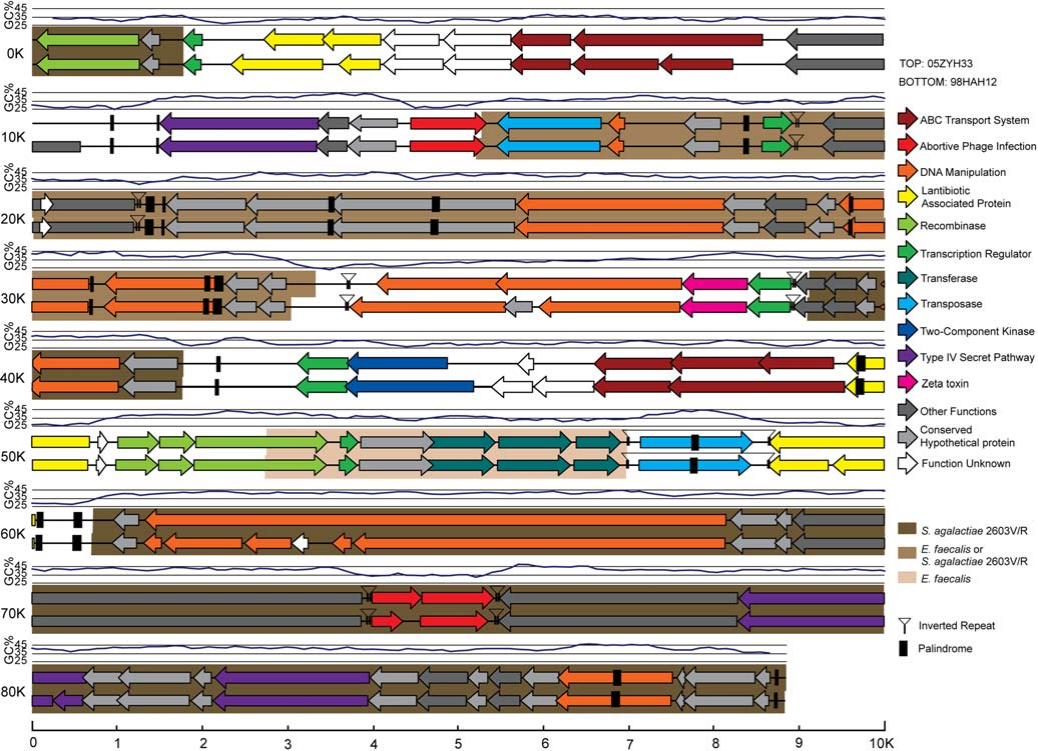
Molecular dissection of 89K, a PAI candidate. Description of the elements with various origins is presented in different colors.

Via the genome-wide display of GC contents, we observed 6 possible GIs with abnormal GC contents, varying in length from 15 kb to 40 kb (Table S3&Fig. 2F). To our surprise, co-linearity comparison of 98HAH12 genome with that of P1/7 shed light on an additional DNA fragment of ∼89 kb in 98HAH12 (Fig. 2A&Fig. 2B). Similarly, the linear genomic comparison between 05ZYH33 and P1/7 also disclosed an additional large DNA fragment of ∼89 kb in 05ZYH33 (Fig. 2A&Fig. 2C). However, the genome of 98HAH12 matched well with that of 05ZYH33 (Fig. 2A&2D). Observing this unexpected DNA fragment designated 89K (Fig. 2E), we speculated that it is possibly a mosaic PAI of ∼89 kb. Notably, 89K seemed to be unique to the Chinese virulent strains (98HAH12&05ZYH33) responsible for STSS (Fig. 2). Further bioinformatics analysis showed that 89K shares an average GC content of 36.8% (Fig. 2F), much lower than the average genomic GC content of ∼41.1% (Table S2). In addition, CHI-square test was applied to probe the difference in codon usage of 89K from that of the whole genome. The resulting p-value is 0.05 (Table S4). Therefore, there is a preference of codon usage in 89K consistent with its foreign acquisition through HGT. Two versions of 89K, separately renamed as 89K_98 _and 89K_05_ in 98HAH12 and 05ZYH33 respectively, are approximately 99% identical, except that 89K_05_ is 19 bp longer than 89K_98_. They encode 67 and 71 potential ORFs, respectively (Fig. 3).

Further analysis of 89K showed clearly that it satisfies nearly all the criteria to be considered as a PAI (Fig. 3): 1) In view of VAFs, we identified a zeta-toxin, three ABC transporter cassettes that play important roles in controlling efflux and influx across cell membranes for small substances, two TCSs with key links to quorum sensing to initiate the infection cycle through regulating efficient bacterial density and adapting to different environments, and three different components of T4SS; 2) 89K, a candidate PAI is present in the two highly virulent strains, 98HAH12 and 05ZYH33, but absent in an avirulent isolate 05HAS68 that has been well confirmed in our laboratory, indicating its potential relationship to high pathogenicity; 3) It possesses ∼89 kb, just falling into the size range from 10 kb to 200 kb required by a representative PAI; 4) It is located adjacent to the 3′-terminus of 50S ribosome gene, a house-keeping gene, similar to tRNA gene in PAIs from Gram negative bacteria; 5) Its low GC content (36.8%) is quite different from that of the core genome (41.1%); 6) There are many different mobile genetic elements such as Tn5252-like homologues, transposases, inverted repeats, recombinases, direct repeats (DR), etc.; 7) Its mosaic architecture allows for homologous genes found in several different bacterial origins including *Enterococcus faecalis* (Table S5&Table S6). In summary, the major features of typical PAIs are present in 89K, strongly supporting our initial hypothesis of it being a specific PAI candidate in Chinese virulent SS2 strains.

### Reproduction of High Pathogenicity in Chinese Virulent Strains

Those piglets infected by both 98HAH12 and 05ZYH33 all died within 30 hrs, and others inoculated with S10, reproduced remarkable signs of severe infection (limping, shivering high fever, CNS failure, and respiratory failure) within 36 hrs, and died at day 7 post-infection. However, the 4 pigs infected with 05HAS68, survived without any obvious symptoms, implying that it is non-virulent. Collectively, the results of pig infection experiments demonstrated clearly that both strains (98HAH12 and 05ZYH33) responsible for STSS are of high virulence, and they seem to share much more invasiveness than the Holland virulent strain S10.

### Specificity of 89K in Chinese SS2 Strains Causing STSS

**Figure 4:**
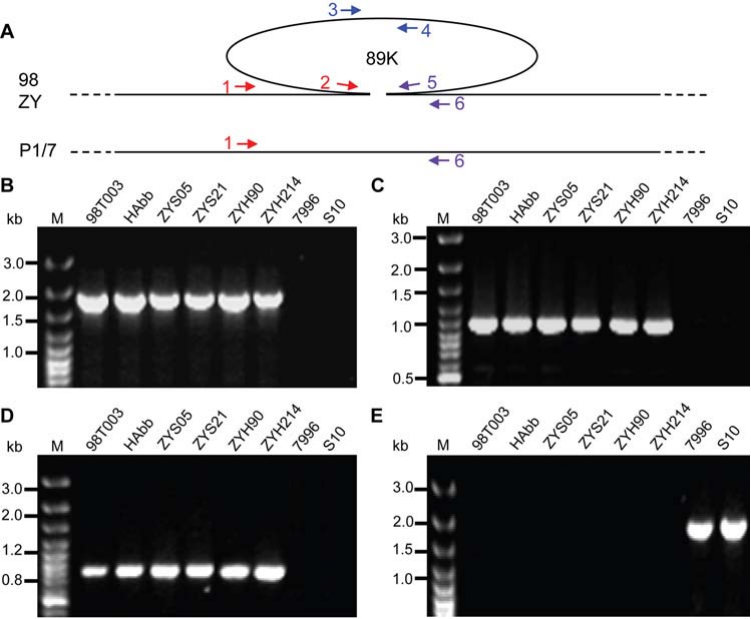
Confirmation of the 89K specificity in highly invasive strains of Chinese SS2 responsible for STSS. (A) Schematic representation of SS2 genomes with 89K and relevant primers highlighted. (B) PCR assay of left border of 89K with primer 1&2. (C) PCR assay of internal gene of 89K with primer 3&4. (D) PCR assay of right border of 89K with primer 5&6. (E) PCR amplification of the complete region spanning 89K with primer 1&6.

To address whether 89K is prevalent and unique among the extremely virulent SS2 strains that are responsible for both large scale outbreaks of severe human SS2 infections in China, we designed three sets of primers to amplify different regions in 89K (Fig. 4A&Table S1). The results showed that 1) both boundary fragments (Left in Fig. 4B and Right in Fig. 4D) and the interior fragment (Fig. 4C) can be amplified in 23 Chinese virulent isolates (Table 2), as exemplified by 98T003, HAbb, ZYS05, ZYS21, ZYH90, and ZYH214 (Figure 4), but fail to be amplified in 10 international SS2 strains, such as avirulent Holland strain 7996 and virulent Holland strain S10 (Fig. 4&Table 2); 2) A ∼1.5 kb PCR product can be obtained in all the tested foreign strains (e.g., 7996 and S10) with two boundary primers located on the adjacent chromosome of 89K, but not in other Chinese virulent isolates (Fig. 4E&Table 2); 3) For the 7 Chinese strains examined (5 are avirulent and 2 are less virulent), PCR results were the same as the aforementioned international strains (Table 2). Combined with the above comparative genomic results derived from 4 strains of SS2 (98HAH12, 05ZYH33, P1/7 and 05HAS68), our results demonstrate clearly that 89K is a novel mosaic PAI candidate and is also prevalent and unique to the Chinese SS2 virulent strains responsible for the two severe outbreaks with a hallmark of STSS in 1998 and 2005. Those strains would be the newly emerging virulent variants of 89K, which is acquired possibly by HGT during the evolution[15], [17], and correlated with STSS and high pathogenicity to some extent[16].

**Table 2:** Statistics of PCR detection for 89K in SS2

Strains	Origins (Years)	Locations	Virulence	89K
***Chinese strains of SS2*** (**30** in total)				
17-19	Healthy swine, 2006	Jiangsu, China	Avirulent	−
05ZYH33*	STSS patient, 2005	Sichuan, China	Highly virulent	+
05ZYH36	STSS patient, 2005	Sichuan, China	Highly virulent	+
ZYH38	STSS patient, 2005	Sichuan, China	Highly virulent	+
ZYH45	STSS patient, 2005	Sichuan, China	Highly virulent	+
ZYH55	STSS patient, 2005	Sichuan, China	Highly virulent	+
ZYH87	STSS patient, 2005	Sichuan, China	Highly virulent	+
ZYH90	STSS patient, 2005	Sichuan, China	Highly virulent	+
ZYH214	STSS patient, 2005	Sichuan, China	Highly virulent	+
ZYH354-1	STSS patient, 2005	Sichuan, China	Highly virulent	+
ZYS05	Died swine, 2005	Sichuan, China	Highly virulent	+
ZYS19	Died swine, 2005	Sichuan, China	Highly virulent	+
ZYS21	Died swine, 2005	Sichuan, China	Highly virulent	+
ZYS22	Died swine, 2005	Sichuan, China	Highly virulent	+
05HAS68*	Healthy swine, 2005	Jiangsu, China	Avirulent	−
05-J2a	Healthy swine, 2005	Jiangsu, China	Avirulent	−
05-J2d	Healthy swine, 2005	Jiangsu, China	Avirulent	−
05-14e	Healthy swine, 2005	Jiangsu, China	Avirulent	−
98HAH12*	STSS patient, 1998	Jiangsu, China	Highly virulent	+
98T003	STSS patient, 1998	Jiangsu, China	Highly virulent	+
HAbb	STSS patient, 1998	Jiangsu, China	Highly virulent	+
98002	STSS patient, 1998	Jiangsu, China	Highly virulent	+
98147#	STSS patient, 1998	Jiangsu, China	Highly virulent	+
98150#	STSS patient, 1998	Jiangsu, China	Highly virulent	+
98145#	Died swine, 1998	Jiangsu, China	Highly virulent	+
98146#	Died swine, 1998	Jiangsu, China	Highly virulent	+
98148#	Died swine, 1998	Jiangsu, China	Highly virulent	+
98151#	Died swine, 1998	Jiangsu, China	Highly virulent	+
S006#	Swine, before 1998	China	Less virulent	−
S008#	Swine, before 1998	China	Less virulent	−
***International strains of SS2*** (**10** in total)				
7996	Swine	Holland	Avirulent	−
S10	Swine	Holland	Highly virulent	−
T15	Swine	Holland	Avirulent	−
8004	Swine	Holland	Highly virulent	−
8011	Swine	Holland	Highly virulent	−
8012	Swine	Holland	Highly virulent	−
8014	Swine	Holland	Highly virulent	−
8019	Swine	Holland	Highly virulent	−
S735	Swine	Canada	Highly virulent	−
SS2-N	Swine	Germany	Highly virulent	−

* the genomes of Chinese SS2 strains have been sequenced.

# those strains were collected in Academy of Military Medical Sciences, P. R. China.

+ being positive in PCR assay for 89K.

− being negative in PCR assay for 89K.

### Preliminary Expression Analysis of 89K

**Figure 5:**
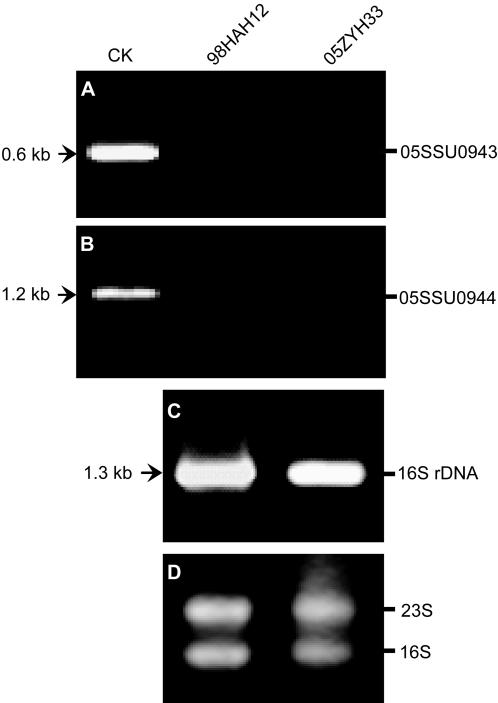
RT-PCR analysis of 89K expression profile exemplified by a TCS. (A) RT-PCR analysis of the response regulator (05SSU0943) at its transcriptional level. The PCR product (05SSU0943) at the position of the arrow (∼0.6 kb), is used as control (CK). (B) RT-PCR analysis of the sensor histidine kinase (05SSU0944) at its transcriptional level. The PCR product (05SSU0944) functions as control (CK) at the position of the arrow (∼0.2 kb). (C) RT-PCR analysis of 16S rRNA, one of the most housekeeping genes featuring the constitutive expressions. The expected fragment is of ∼1.3 kb at the arrow position. (D) Electrophoresis analysis of the total RNA isolated from both representative SS2 strains (98HAH12&05ZYH33). Two conservative subunits of bacterial rRNAs are pointed out with 16S and 23S, respectively.

Like other VAFs, those genes dispersed in PAIs were suggested to be not constitutively expressed but to respond to environmental conditions[15], [17], [19]. In light of this point, we selected a TCS as an indicator of expression profile of 89K. This TCS comprised 2 components, a DNA-binding response regulator (05SSU0943) and a sensor histidine kinase (05SSU0944). The quality of RNA preparations was confirmed by the presence of bands corresponding to 23S and 16S rRNA (Fig. 5C&Fig. 5D). However, we observed that neither 05SSU0943 (∼0.6 kb) nor 05SSU0944 (∼1.2 kb) could be amplified by RT-PCR (Fig. 5A&Fig. 5B), which may in turn support the previous postulate that their expression may be involved in enviramental regulation. One possible explanation for our data here may be that the in vitro growth conditions differ from those found in the host.

## DISCUSSION

*S. suis* contains 35 different serotypes that are officially determined on the basis of the different capsule antigens[1], [2]. SS2 is the most frequently isolated from clinically infected pigs worldwide, among which the virulence can be categorized into highly pathogenic, hypovirulent and avirulent[1]–[3]. Furthermore, SS2 has been regarded as one of the major zoonotic pathogens that cause sporadic cases of meningitis and sepsis in humans[3].

To date, most reported cases of human STSS were associated with GAS in *Streptococcus* [8]–[10], [12], while STSS due to non-GAS streptococci is relatively limited[13]. Two newly confirmed SS2 outbreaks in China, one in 1998 and the other in 2005, triggered human STSS on a large scale, implying that SS2 is another aetiological pathogen for STSS. However, the mitogenic activity in these highly invasive isolates were not observed by Tang et al.[3], suggesting that some different molecular mechanism is possibly the answer for STSS due to SS2 in China. Very recently, Brandt et al. also reported a similar finding that mitogenicity cannot be detected even in speG- and speG(dys)-positive *Streptococcus dysgalactiae* subspecies equisimilis isolates from patients with invasive infections[42]. Thus, the highly invasive infection (esp. STSS) may not be only associated with super-antigens, but also involve other factors.

Luckily, with the aid of comparative genomics, we observed that 89K, a PAI-like island, presents some difference between 98HAH12 and 05ZYH33. As it has been documented that ABC-transporters may be connected with a PAI in UPEC[15], [17], 89K possesses several virulence-associated components that are related to ABC-transporters, T4SS, etc. besides the general elements such as transposases and palindrome sequences. Moreover, the mosaic structure of 89K can be characterized with different origins of bacterial pathogens (Fig. 3, Table S5&Table S6), indicating frequent HGT in their coexisting niches.

In conclusion, we report here a unique element, 89K, in Chinese virulent strains, representing the first PAI candidate from *S. suis* worldwide. It may provide a new glimpse of STSS caused by Chinese SS2 strains at the genomic level, and facilitate understanding on the molecular mechanism that modulates the high pathogenicity and STSS triggered by Chinese SS2 virulent strains. Furthermore, the introduction of PAI into SS2 will attract much attention worldwide, open up another research era and need further functional verification.

## Supporting Information

Protocol S1 Detailed Materials and Methods
(0.09 MB DOC)

Table S1 Primers used for 89K PCR detection
(0.03 MB DOC)

Table S2 General features of three SS2 genomes
(0.05 MB DOC)

Table S3 Outline of the potential genomic islands in SS2
(0.03 MB DOC)

Table S4 Comparison of codon usage between the whole genome and 89K
(0.08 MB DOC)

Table S5 Uncompleted statistics of possible origins in 89K98
(0.12 MB DOC)

Table S6 Uncompleted statistics of possible origins in 89K05
(0.11 MB DOC)
