# Supplementary material for: A Glimpse of Streptococcal Toxic Shock Syndrome from Comparative Genomics of S. suis 2 Chinese Isolates
Source: PLoS One. 2007 Mar 21;2(3):e315. doi: 10.1371/journal.pone.0000315 (PMC1820848; doi:10.1371/journal.pone.0000315)
Supplement: Protocol S1 — Detailed Materials and Methods (0.09 MB DOC) [file pone.0000315.s001.doc]

**Protocol S1. Detailed Materials and Methods**

***Bacterial strains and growth.*** All the 40 SS2 strains used in this study were firstly plated on Columbia agar medium containing 6% (vol/vol) sheep blood to characterize their haemolytic types[1]. Subsequently, they were cultivated in Todd-Hewitt broth (THB, code CM189; Oxoid, Basingstock, Hampshire, United Kingdoms) for preparation of genomic DNA[1]., Three strains of Chinese SS2 (98HAH12, 05ZYH33 & 05HAS68) were selected for genome sequencing. In brief, both 98HAH12 (a human isolate from Haian County, Jiangsu Province, 1998) and 05ZYH33 (a human isolate in Ziyang County, Sichuan Province, 2005) were representative of highly pathogenic SS2 isolated from fatal cases of STSS[1]. 05HAS68 (a healthy swine isolate in Haian County, Jiangsu Province, 2005) was an avirulent strain of SS2 [1].

***Molecular manipulations*.** Bacterial genomic DNA (98HAH12, 05ZYH33 & 05HAS68) was prepared as described by Tang et al.[1], and then genomic libraries were constructed and evaluated according to standard procedures. Briefly, sheared DNA samples were fractionated to construct two different genomic libraries, containing average insert sizes of 1.5~3.0kb and 4.0~5.0kb, respectively. The resulting pUC18-derived libratory plasmids were extracted using the alkaline lysis method and subjected to direct DNA sequencing with automated capillary DNA sequencers (ABI3730 or MegaBACE1000)[1,2].

***Genome sequencing and assembly*.** In total, raw sequences of the three bacterial isolates (98HAH12, 05ZYH33 & 05HAS68) gave rise to 22395, 20731 and 26858 sequencing reads respectively, which were assembled into contigs by utilizing the software package of Phred-Phrap-Consed[3-5],. which resulted in ~12-fold, ~12-fold and nearly 8-fold genome coverage, respectively. PCR amplifications were utilized to close gaps. Genome sequences have been deposited in GenBank.

***Gene annotation and comparative genomics*.** Gene annotation was completed using routine methods. Putative ORFs were predicted with Glimmer[6] and their functions were annotated with blastP (e-value <1e-10, 80% identity, and 100 amino acids in overlapped length) and information from GenBank and UniProt (version 47). Secondly, they were categorized based on Interpro, GO, and COG (Clusters of Orthologous Genes). Finally, functional pathways were annotated based on KEGG[7], tRNA genes and repeats were predicted with tRNAscan-SE[8] and Repeatmasker (<http://repeatmasker.genome.washington.edu/cgi-bin/RMZ.pl>). The software tool (Mummer[9]) was used for genome comparisons.

***SNP analysis.*** SNPs among the three SS2 genomes were detected by BLSTNB (e<10^-5^). Synonymous and nonsynonymous sites were determined by ClustalW[2]. The software of YN00[10] combined with PAML package [11] was utilized for Ka and Ks calculations, resulting in all the relevant numerical values.

***Prediction of the origins and termini in S. suis genomes*.** We applied the program ORIGINX[12] to predict the potential origin and terminus in each *S. suis* genome. We used the default parameters: window size = 90%, step = 1000bp; threshold = 40bits and oligo-length = 6 and the weighting schemes of G/C and A/T are as follows: G/C where the weights are +2 for G, -2 for C and 0 for A and T; and A/T where the weights are +2 for A, -2 for T and 0 for G and C.

***Calculation of the GC contents of S. suis genomes*.** The GC contents of 98HAH12, 05ZYH33, and P1/7 were calculated by using the program INFOSEQ in the EMBOSS package[13]. First, we used 20 kb windows with 5kb overlap for the whole genomes, and then we set artificially ~89 kb gaps into P1/7 at the position where the corresponding segments reside in 98HAH12 and 05ZYH33. Second, we used 500 bp windows overlapped by 100 bp to compute the G+C% on the ~89 kb segments observed only in 98HAH12 and 05ZYH33.

***Identification of putative genomic islands in SS2*.** To identify possible genomic islands (GIs) in *S. suis* genomes, we adopted the steps below: First, GC content was determined as above. Second, the following stringent criteria were employed: 1) The putative GI width must be over 15 kb; 2) the G+C% of the potential GI must have over 4.5 differences greater or smaller than the G+C% of *S. suis* P1/7. To determine possible sources of horizontally transferred GIs, we performed BLASTN. If the hit nucleotide sequence had >80% identity with the possible GI and matched 80% in length, we considered it as the potential source.

***Characterization of 89K*.** First, the GC contents of 89K (in 98HAH12 & 05ZYH33) were determined as described above. Second, functional annotation was conducted with the aid of BLASTN. Third, both programs (EINVERTED and PALINDROME in EMBOSS package[13]) were used to predict the inverted repeats in 89K. In detail, the parameters are as follows: EINVERTED where the gap penalty = 12, minimum score threshold = 50, match = 3 and mismatch = 4; and PALINDROME where the minimum length = 10, maximum length = 100, maximum gap = 100 and number of mismatch = 0. The codon usage between genome and 89K regions was compared by CHI-square test.

***Preliminary expression analysis of PAI*.** Two representatives of highly pathogenic SS2 Chinese strains (98HAH12 & 05ZYH33) were cultivated for the extraction of total RNA. Bacterial RNA was extracted from 5 ml culture using Trizol reagents (GIBCO-BRL), and reverse transcription was performed using random primers according to manufacturer’s instructions.. Here, a specific TCS consisting of 2 members (named temporarily 05SSU0943 and 05SSU0944) in 89K was selectively used to monitor the transcriptional level of the candidate PAI. Therefore, 2 sets of specific primers (0943-F: 5’-TTG AAA ATT TTA TTA ATA GAT GAT C-3’; 0943-R: 5’-TTA CTT AAT ATA TCC TAA CTT CTG-3’; 0944-F: 5’-TTG TTT TTT CAA AAG TTA CAG AC-3’ and 0944-R: 5’-T TA TCT ATT AAT AAA ATT TTC AAT TGC C -3’) were designed. Moreover, as a positive control for RT-PCR, one pair of conserved primers for 16S rDNA (16S-F: 5’-TAA CAG TAT TTA CCG CAT G-3’ and 16S-R: 5’-TAC CTT GTT ACG ACT TCA-3’) was also used as a reference. Subsequently, PCR was conducted to amplify the above 3 target DNA fragments (05SSU0943, 05SSU0944 and 16S rDNA). The PCR program consisted of pre-incubation for 5min at 95°C, followed by 30cycles of denaturation for 0.5min at 95°C, primer annealing for 0.5min at 50°C, and extension for 1.0min at 72°C. The final extension step was kept for 8min at 72°C. Finally, the PCR products were separated by electrophoresis in 1.0% agarose gels (Brazil) supplemented with 0.2% ethidium bromide (EB).

**Supporting References**

1. Tang J, Wang C, Feng Y, Yang W, Song H, et al. (2006) Streptococcal toxic shock syndrome caused by Streptococcus suis serotype 2. PLoS Med 3: e151: 0668-0676.

2. Qian W, Jia Y, Ren SX, He YQ, Feng JX, et al. (2005) Comparative and functional genomic analyses of the pathogenicity of phytopathogen Xanthomonas campestris pv. campestris. Genome Res 15: 757-767.

3. Ewing B, Green P (1998) Base-calling of automated sequencer traces using phred. II. Error probabilities. Genome Res 8: 186-194.

4. Ewing B, Hillier L, Wendl MC, Green P (1998) Base-calling of automated sequencer traces using phred. I. Accuracy assessment. Genome Res 8: 175-185.

5. Gordon D, Abajian C, Green P (1998) Consed: a graphical tool for sequence finishing. Genome Res 8: 195-202.

6. Delcher AL, Harmon D, Kasif S, White O, Salzberg SL (1999) Improved microbial gene identification with GLIMMER. Nucleic Acids Res 27: 4636-4641.

7. Kanehisa M, Goto S, Hattori M, Aoki-Kinoshita KF, Itoh M, et al. (2006) From genomics to chemical genomics: new developments in KEGG. Nucleic Acids Res 34: D354-357.

8. Lowe TM, Eddy SR (1997) tRNAscan-SE: a program for improved detection of transfer RNA genes in genomic sequence. Nucleic Acids Res 25: 955-964.

9. Delcher AL, Phillippy A, Carlton J, Salzberg SL (2002) Fast algorithms for large-scale genome alignment and comparison. Nucleic Acids Res 30: 2478-2483.

10. Yang Z, Nielsen R (2000) Estimating synonymous and nonsynonymous substitution rates under realistic evolutionary models. Mol Biol Evol 17: 32-43.

11. Yang Z (1997) PAML: a program package for phylogenetic analysis by maximum likelihood. Comput Appl Biosci 13: 555-556.

12. Worning P, Jensen LJ, Hallin PF, Staerfeldt HH, Ussery DW (2006) Origin of replication in circular prokaryotic chromosomes. Environ Microbiol 8: 353-361.

13. Rice P, Longden I, Bleasby A (2000) EMBOSS: the European Molecular Biology Open Software Suite. Trends Genet 16: 276-277.
