# Supplementary material for: A Glimpse of Streptococcal Toxic Shock Syndrome from Comparative Genomics of S. suis 2 Chinese Isolates
Source: PLoS One. 2007 Mar 21;2(3):e315. doi: 10.1371/journal.pone.0000315 (PMC1820848; doi:10.1371/journal.pone.0000315)
Supplement: Table S1 — Primers used for 89K PCR detection (0.03 MB DOC) [file pone.0000315.s002.doc]

**Table S1. Primers used for 89K PCR detection**

| Codes of the primers | Primer sequences | Expected size (kb) |
| --- | --- | --- |
| 1 | 5’-CAC GCA TCT CGT AGA GTT TGA C-3’ | ~1.8 (1&2)* |
| 2 | 5’-AGA TTG CGA GGC TTT TAG ATT G-3’ |
| 3 | 5’-TCG CCA CTA TGG TAT CTG CTT A-3’ | ~1.0 (3&4)* |
| 4 | 5’-GAT TGT GGA CCA TGC TGT TTA G-3’ |
| 5 | 5’-ATA AAT AGC CCC ATC CTC ATC A-3’ | ~0.7 (5&6)*  ~1.5 (1&6)* |
| 6 | 5’-GCG TAG CTG CTT AGT GCT ACA A-3’ |

*, the PCR experiments were carried out following the primer combinations (1&2, 3&4, 5&6 and 1&6).
