# Supplementary material for: A Glimpse of Streptococcal Toxic Shock Syndrome from Comparative Genomics of S. suis 2 Chinese Isolates
Source: PLoS One. 2007 Mar 21;2(3):e315. doi: 10.1371/journal.pone.0000315 (PMC1820848; doi:10.1371/journal.pone.0000315)
Supplement: Table S2 — General features of three SS2 genomes (0.05 MB DOC) [file pone.0000315.s003.doc]

**Table S2.** General features of three SS2 genomes

| **Organization and function characterization**  **of the genomes** | **Strains** | | |
| --- | --- | --- | --- |
|  | **98HAH12** | **05ZYH33** | **P1/7** |
| **General features** | | | |
| Genome size (bp) | 2,095,720 | 2,096,331 | 2,007,491 |
| GC content (%) | 41.11 | 41.11 | 41.30 |
| Total number of predicted ORFs | 2191 | 2194 | 1969 |
| General function prediction only | 176 | 180 | 167 |
| RNA | 69 | 69 | 69 |
| **Function category** | | | |
| Amino acid transport and metabolism | 136 | 146 | 129 |
| Carbohydrate transport and metabolism | 174 | 177 | 158 |
| Inorganic ion transport and metabolism | 78 | 76 | 70 |
| Coenzyme transport and metabolism | 48 | 47 | 45 |
| Secondary metabolites biosynthesis, transport  and catabolism | 12 | 12 | 11 |
| Energy production and conversion | 50 | 48 | 46 |
| Replication, recombination and repair | 148 | 152 | 138 |
| Transcription | 132 | 125 | 118 |
| Translation, ribosomal structure and biogenesis | 149 | 154 | 144 |
| Posttranslational modification, protein turnover, chaperones | 54 | 53 | 49 |
| Intracellular trafficking, secretion, and vesicular transport | 22 | 21 | 17 |
| Cell motility | 7 | 6 | 6 |
| Cell wall/membrane/envelope biogenesis | 95 | 94 | 90 |
| Cell cycle control, cell division, chromosome  partitioning | 24 | 25 | 23 |
