# Supplementary material for: A Glimpse of Streptococcal Toxic Shock Syndrome from Comparative Genomics of S. suis 2 Chinese Isolates
Source: PLoS One. 2007 Mar 21;2(3):e315. doi: 10.1371/journal.pone.0000315 (PMC1820848; doi:10.1371/journal.pone.0000315)
Supplement: Table S3 — Outline of the potential genomic islands in SS2 (0.03 MB DOC) [file pone.0000315.s004.doc]

**Table S3.** Outline of the potential genomic islands in SS2

| No. | Approximate position (kb) | GC % | ΔGC % | Category of the main components |
| --- | --- | --- | --- | --- |
| 1 | 195~210 | 34.1 | -6.2 | Metabolisms & ABC transport system |
| 2 | 270~310 | 45.9 | +4.6 | ABC transport system，  Transcription regulator & Enzymes |
| 3 | 555~585 | 35.6 | -5.2 | Transposases & Insertion sequence |
| 4 | 830~855 | 34.2 | -7.1 | Unknown |
| 5 | 1010~1025 | 36.4 | -4.9 | Sugar metabolism |
| 6 | 1220~1235 | 36.5 | -4.8 | polysaccharide biosynthesis |
