# Supplementary material for: A Glimpse of Streptococcal Toxic Shock Syndrome from Comparative Genomics of S. suis 2 Chinese Isolates
Source: PLoS One. 2007 Mar 21;2(3):e315. doi: 10.1371/journal.pone.0000315 (PMC1820848; doi:10.1371/journal.pone.0000315)
Supplement: Table S4 — Comparison of codon usage between the whole genome and 89K (0.08 MB DOC) [file pone.0000315.s005.doc]

**Table S4.** Comparison of codon usage between the whole genome and 89K

| **Codon** | **Genome** | **89K** | **Codon** | **Genome** | **89K** | **Codon** | **Genome** | **89K** | **Codon** | **Genome** | **89K** |
| --- | --- | --- | --- | --- | --- | --- | --- | --- | --- | --- | --- |
| UUU | 30.0(17597) | 36.6(940) | UCU | 14.7(8648) | 15.3(393) | UAU | 24.3(14226) | 32.0(824) | UGU | 4.1(2388) | 4.8(123) |
| UUC | 15.2(8937) | 11.1(286) | UCC | 6.8(3977) | 7.8(200) | UAC | 13.4(7873) | 12.1(312) | UGC | 1.9(1131) | 2.1(54) |
| UUA | 18.1(10616) | 31.1(799) | UCA | 13.8(8069) | 17.8(457) | UAA | 0.0(0) | 0.0(0) | UGA | 0.0(0) | 0.0(0) |
| UUG | 31.5(18480) | 21.5(553) | UCG | 4.7(2742) | 4.4(112) | UAG | 0.0(0) | 0.0(0) | UGG | 9.2(5415) | 8.1(209) |
|  |  |  |  |  |  |  |  |  |  |  |  |
| CUU | 17.4(10227) | 18.0(462) | CCU | 10.1(5940) | 9.9(254) | CAU | 11.7(6845) | 12.4(320) | CGU | 16.8(9854) | 11.3(291) |
| CUC | 12.4(7270) | 9.3(240) | CCC | 3.5(2044) | 3.9(100) | CAC | 7.2(4241) | 4.2(108) | CGC | 7.6(4433) | 4.4(114) |
| CUA | 11.5(6742) | 14.5(374) | CCA | 15.8(9279) | 12.1(312) | CAA | 25.1(14704) | 28.6(735) | CGA | 5.2(3033) | 8.6(220) |
| CUG | 11.3(6611) | 11.1(286) | CCG | 4.6(2682) | 3.9(101) | CAG | 17.2(10109) | 16.5(425) | CGG | 4.0(2372) | 2.8(73) |
|  |  |  |  |  |  |  |  |  |  |  |  |
| AUU | 41.4(24274) | 43.0(1105) | ACU | 15.7(9230) | 16.1(413) | AAU | 28.3(16591) | 36.9(949) | AGU | 12.3(7241) | 17.7(456) |
| AUC | 23.3(13668) | 14.7(379) | ACC | 14.4(8460) | 15.2(391) | AAC | 15.0(8793) | 13.2(340) | AGC | 8.6(5067) | 7.6(196) |
| AUA | 7.0(4076) | 17.3(445) | ACA | 20.1(11772) | 20.0(515) | AAA | 38.9(22840) | 48.2(1240) | AGA | 5.7(3369) | 13.8(354) |
| AUG | 24.3(14278) | 22.1(569) | ACG | 7.6(4450) | 7.7(197) | AAG | 24.3(14245) | 25.9(667) | AGG | 2.3(1361) | 4.0(104) |
|  |  |  |  |  |  |  |  |  |  |  |  |
| GUU | 26.3(15406) | 19.8(509) | GCU | 28.0(16424) | 18.2(467) | GAU | 36.4(21364) | 40.5(1042) | GGU | 29.3(17158) | 16.8(432) |
| GUC | 16.3(9536) | 12.9(332) | GCC | 16.9(9933) | 11.2(288) | GAC | 19.1(11177) | 18.0(464) | GGC | 11.8(6924) | 9.6(248) |
| GUA | 14.8(8702) | 15.3(394) | GCA | 22.9(13451) | 15.6(400) | GAA | 49.1(28771) | 51.5(1325) | GGA | 17.1(10045) | 19.6(505) |
| GUG | 13.4(7884) | 14.2(366) | GCG | 9.5(5562) | 6.0(155) | GAG | 22.2(13047) | 22.1(567) | GGG | 8.3(4861) | 8.6(220) |

The p-value is 0.05 by CHI-square test. Thus, obvious difference of the condon usages between the whole genome and 89K was highlighted in yellow.
