# Supplementary material for: A Glimpse of Streptococcal Toxic Shock Syndrome from Comparative Genomics of S. suis 2 Chinese Isolates
Source: PLoS One. 2007 Mar 21;2(3):e315. doi: 10.1371/journal.pone.0000315 (PMC1820848; doi:10.1371/journal.pone.0000315)
Supplement: Table S5 — Uncompleted statistics of possible origins in 89K98 (0.12 MB DOC) [file pone.0000315.s006.doc]

**Table S5.** Uncompleted statistics of possible origins in 89K98

| **CDS** | **Identity (%)** | **Possible Origins** | **Function Assignments** |
| --- | --- | --- | --- |
| 98SSU0924 | 100 | *Enterococcus faecalis* | Excisionase of Tn1545 or Tn916 |
| 98SSU0925 | 100 | *Enterococcus faecalis* | ORF7 |
| 98SSU0933 | 100 | *Enterococcus faecalis* | Hypothetical protein in Tn916_08 |
| 98SSU0935 | 100 | *Enterococcus faecalis* | Hypothetical protein in Tn916_06 |
| 98SSU0939 | 100 | *Enterococcus faecalis* | Hypothetical protein in Tn916_02 |
| 98SSU0964 | 100 | *Enterococcus faecalis* | Putative aminoglycoside 6-adenylyltansferase |
| 98SSU0965 | 100 | *Enterococcus faecalis* | Putative adenine phosphoribosyltransferase |
| 98SSU0932 | 97 | *Enterococcus faecalis* | ATPase involved in DNA repair |
| 98SSU0938 | 97 | *Enterococcus faecalis* | Hypothetical protein Tn916_03 |
| 98SSU0937 | 96 | *Enterococcus faecalis* | Hypothetical protein Tn916_04 |
| 98SSU0930 | 95 | *Enterococcus faecalis* | Hypothetical protein Tn916_11 |
| 98SSU0934 | 90 | *Enterococcus faecalis* | Hypothetical protein Tn916_07 |
| 98SSU0928 | 100 | *Enterococcus faecium* | Translation elongation factors (GTPases) |
| 98SSU0963 | 94 | *Enterococcus faecium* | SAM-dependent methyltransferases |
| 98SSU0962 | 87 | *Enterococcus faecium* | Hypothetical protein |
| 98SSU0966 | 82 | *Enterococcus faecium* | putative transposase |
| 98SSU0923 | 100 | *Streptococcus agalactiae* 2603V/R | Tn916, transposase |
| 98SSU0926 | 100 | *Streptococcus agalactiae* 2603V/R | Putative transcriptional regulator |
| 98SSU0936 | 99 | *Streptococcus agalactiae* 2603V/R | Putative transcriptional regulator |
| 98SSU0908 | 98 | *Streptococcus agalactiae* 2603V/R | Site-specific recombinase |
| 98SSU0909 | 98 | *Streptococcus agalactiae* 2603V/R | Hypothetical protein SAG1248 |
| 98SSU0974 | 98 | *Streptococcus agalactiae* 2603V/R | SNF2 family protein |
| 98SSU0929 | 95 | *Streptococcus agalactiae* 2603V/R | Hypothetical protein |
| 98SSU0931 | 95 | *Streptococcus agalactiae* 2603V/R | Putative membrane protein |
| 98SSU0993 | 95 | *Streptococcus agalactiae* 2603V/R | C-5 cytosine-specific DNA methylase |
| 98SSU0947 | 93 | *Streptococcus agalactiae* 2603V/R | Hypothetical protein SAG1275 |
| 98SSU0978 | 92 | *Streptococcus agalactiae* 2603V/R | Agglutinin receptor |
| 98SSU0975 | 91 | *Streptococcus agalactiae* 2603V/R | SNF2 family protein |
| 98SSU0981 | 87 | *Streptococcus agalactiae* 2603V/R | Orf28 |
| 98SSU0986 | 83 | *Streptococcus agalactiae* 2603V/R | Hypothetical protein SAG1290 |
| 98SSU0989 | 80 | *Streptococcus agalactiae* 2603V/R | Putative protease |
| 98SSU0944 | 99 | *Streptococcus suis* 89/1591 | Predicted transcriptional regulators |
| 98SSU0977 | 98 | *Streptococcus suis* 89/1591 | Hypothetical protein |
| 98SSU0987 | 98 | *Streptococcus suis* 89/1591 | Type IV secretory pathway, VirD4 components |
| 98SSU0946 | 97 | *Streptococcus suis* 89/1591 | Methyl-accepting chemotaxis protein |
| 98SSU0972 | 97 | *Streptococcus suis* 89/1591 | DNA methylase |
| 98SSU0980 | 97 | *Streptococcus suis* 89/1591 | Uncharacterized conserved protein |
| 98SSU0984 | 97 | *Streptococcus suis* 89/1591 | Hypothetical protein |
| 98SSU0990 | 97 | *Streptococcus suis* 89/1591 | Hypothetical protein |
| 98SSU0992 | 97 | *Streptococcus suis* 89/1591 | Hypothetical protein |
| 98SSU0971 | 96 | *Streptococcus suis* 89/1591 | DNA methylase |
| 98SSU0991 | 96 | *Streptococcus suis* 89/1591 | Arsenate reductase and related proteins, glutaredoxin family |
| 98SSU0949 | 95 | *Streptococcus suis* 89/1591 | Hypothetical protein |
| 98SSU0979 | 95 | *Streptococcus suis* 89/1591 | Predicted transcriptional regulator |
| 98SSU0982 | 95 | *Streptococcus suis* 89/1591 | Type IV secretory pathway, VirB4 components |
| 98SSU0943 | 92 | *Streptococcus suis* 89/1591 | Signal recognition particle GTPase |
| 98SSU0996 | 92 | *Streptococcus suis* 89/1591 | Hypothetical protein |
| 98SSU0948 | 90 | *Streptococcus suis 89/1591* | DNA primase |
| 98SSU0988 | 89 | Streptococcus suis 89/1591 | Hypothetical protein |
| 98SSU0969 | 88 | *Streptococcus suis* 89/1591 | Hypothetical protein |
| 98SSU0995 | 87 | *Streptococcus suis* 89/1591 | Hypothetical protein |
| 98SSU0945 | 85 | *Streptococcus suis* 89/1591 | ATPases with chaperone activity, ATP-binding subunit |
| 98SSU0976 | 85 | *Streptococcus suis* 89/1591 | Hypothetical protein |
| 98SSU0983 | 84 | *Streptococcus suis* 89/1591 | Type IV secretory pathway, VirB4 components |
| 98SSU0985 | 79 | *Streptococcus suis* 89/1591 | ABC-type cobalt transport system |
| 98SSU0920 | 75 | *Streptococcus agalactiae* NEM316 | Unknown |
| 98SSU0919 | 65 | *Streptococcus suis* 89/1591 | Chromosome segregation ATPases |
| 98SSU0910 | 63 | *Streptococcus agalactiae* 2603V/R | Transcriptional regulator, Cro/CI family |
| 98SSU0921 | 62 | *Streptococcus pneumoniae* R6 | Hypothetical protein spr0955 |
| 98SSU0960 | 61 | *Bacillus cereus* ATCC 10987 | Putative DNA recombinase |
| 98SSU0912 | 54 | *Lactococcus lactis* | Nisin biosynthesis regulator, NisR |
| 98SSU0970 | 50 | *Streptococcus agalactiae* 2603V/R | SNF2 family protein |
| 98SSU0915 | 48 | *Corynebacterium efficiens* YS-314 | Putative transport ATP-binding protein |
| 98SSU0950 | 47 | *Bacillus cereus* G9241 | Putative DNA-binding response regulator |
| 98SSU0959 | 44 | *Clostridium thermocellum* ATCC 27405 | Recombinase |
| 98SSU0916 | 43 | *Leifsonia xyli* subsp. xyli str. CTCB07 | ABC transporter, NBP/MSD fusion protein |
| 98SSU0942 | 41 | *Bacillus clausii* KSM-K16 | Hypothetical protein |
| 98SSU0940 | 38 | *Bacillus clausii* KSM-K16 | DNA helicase |
| 98SSU0941 | 38 | *Bacillus clausii* KSM-K16 | Hypothetical protein |
| 98SSU0954 | 38 | *Clostridium difficile* | Cdd4 |
| 98SSU0958 | 38 | *Bacillus cereus* ATCC 10987 | DNA recombinase |
| 98SSU0951 | 32 | *Bacillus thuringiensis* serovar konkukian str. 97-27 | Possible two-component sensor histidine kinase |
| 98SSU0967 | 31 | *Bacillus halodurans* C-125 | Lantibiotic mersacidin modifying enzyme |
| 98SSU0917 | 29 | *Leifsonia xyli* subsp. xyli str. CTCB07 | ABC transporter, NBP/MSD fusion protein |
| 98SSU0956 | 29 | *Staphylococcus aureus* | Lantibiotic modifying enzyme |
| 98SSU0911 | 27 | *Lactococcus lactis* | NisK |
| 98SSU0922 | 27 | *Streptococcus thermophilus* | Putative Abi-alpha protein |
| 98SSU0968 | 27 | *Streptococcus salivarius* | SalB |
| 98SSU0918 | 24 | *Streptomyces avermitilis* MA-4680 | Putative asparagine synthetase |
| 98SSU0955 | 24 | *Staphylococcus aureus* | Transporter |
