# Supplementary material for: A Glimpse of Streptococcal Toxic Shock Syndrome from Comparative Genomics of S. suis 2 Chinese Isolates
Source: PLoS One. 2007 Mar 21;2(3):e315. doi: 10.1371/journal.pone.0000315 (PMC1820848; doi:10.1371/journal.pone.0000315)
Supplement: Table S6 — Uncompleted statistics of possible origins in 89K05 (0.11 MB DOC) [file pone.0000315.s007.doc]

**Table S6**. Uncompleted statistics of possible origins in 89K05

| **CDS** | **Identity (%)** | **Possible origins** | **Function Assignments** |
| --- | --- | --- | --- |
| 05SSU0918 | 100 | *Enterococcus faecalis* | Excisionase from transposon Tn1545 or Tn916 |
| 05SSU0919 | 100 | *Enterococcus faecalis* | ORF7 |
| 05SSU0927 | 100 | *Enterococcus faecalis* | Hypothetical protein in Tn916_08 |
| 05SSU0929 | 100 | *Enterococcus faecalis* | Hypothetical protein in Tn916_06 |
| 05SSU0932 | 100 | *Enterococcus faecalis* | Hypothetical protein in Tn916_03 |
| 05SSU0933 | 100 | *Enterococcus faecalis* | Hypothetical protein in Tn916_02 |
| 05SSU0957 | 100 | *Enterococcus faecalis* | Putative aminoglycoside 6-adenylyltansferase |
| 05SSU0958 | 100 | *Enterococcus faecalis* | Putative adenine phosphoribosyltransferase |
| 05SSU0926 | 97 | *Enterococcus faecalis* | Pypothetical protein (ORF16) Tn916_09 |
| 05SSU0931 | 96 | *Enterococcus faecalis* | DNA segregation ATPase FtsK/SpoIIIE and related proteins |
| 05SSU0924 | 95 | *Enterococcus faecalis* | ORF14, NLP/P60 family protein |
| 05SSU0928 | 90 | *Enterococcus faecalis* | Hypothetical protein Tn916_07 |
| 05SSU0922 | 100 | *Enterococcus faecium* | Translation elongation factors (GTPases) |
| 05SSU0956 | 94 | *Enterococcus faecium* | Methyltransferas |
| 05SSU0955 | 88 | *Enterococcus faecium* | Hypothetical protein |
| 05SSU0959 | 82 | *Enterococcus faecium* | Putative transposase |
| 05SSU0917 | *100* | *Streptococcus agalactiae* 2603V/R | Transposase in Tn916 |
| 05SSU0920 | 100 | *Streptococcus agalactiae* 2603V/R | Putative transcriptional regulator in Tn916 |
| 05SSU0930 | 99 | *Streptococcus agalactiae* 2603V/R | Putative transcriptional regulator in Tn916 |
| 05SSU0903 | 98 | *Streptococcus agalactiae* 2603V/R | Site-specific recombinase, phage integrase family |
| 05SSU0904 | 98 | *Streptococcus agalactiae* 2603V/R | Hypothetical protein SAG1248 |
| 05SSU0923 | 95 | *Streptococcus agalactiae* 2603V/R | Hypothetical protein in Tn916 |
| 05SSU0925 | 95 | *Streptococcus agalactiae* 2603V/R | Putative membrane protein |
| 05SSU0979 | 95 | *Streptococcus agalactiae* 2603V/R | C-5 cytosine-specific DNA methylase |
| 05SSU0940 | 93 | *Streptococcus agalactiae* 2603V/R | Hypothetical protein SAG1275 |
| 05SSU0965 | 92 | *Streptococcus agalactiae* 2603V/R | Agglutinin receptor |
| 05SSU0962 | 91 | *Streptococcus agalactiae* 2603V/R | SNF2 family protein |
| 05SSU0968 | 87 | *Streptococcus agalactiae* 2603V/R | Tn5252, Orf28 |
| 05SSU0972 | 80 | *Streptococcus agalactiae* 2603V/R | Hypothetical protein SAG1290 |
| 05SSU0975 | 80 | *Streptococcus agalactiae* 2603V/R | Putative protease |
| 05SSU0937 | 99 | *Streptococcus suis* 89/1591 | Predicted transcriptional regulators |
| 05SSU0964 | 98 | *Streptococcus suis* 89/1591 | Hypothetical protein |
| 05SSU0973 | 98 | *Streptococcus suis* 89/1591 | Type IV secretory pathway, VirD4 components |
| 05SSU0976 | 98 | *Streptococcus suis* 89/1591 | Hypothetical protein |
| 05SSU0939 | 97 | *Streptococcus suis* 89/1591 | Methyl-accepting chemotaxis protein |
| 05SSU0967 | 97 | *Streptococcus suis* 89/1591 | Uncharacterized conserved protein |
| 05SSU0970 | 97 | *Streptococcus suis* 89/1591 | Hypothetical protein |
| 05SSU0978 | 97 | *Streptococcus suis* 89/1591 | Hypothetical protein |
| 05SSU0966 | 96 | *Streptococcus suis* 89/1591 | Predicted transcriptional regulator |
| 05SSU0977 | 96 | *Streptococcus suis* 89/1591 | Arsenate reductase and related proteins, glutaredoxin family |
| 05SSU0942 | 95 | *Streptococcus suis* 89/1591 | Hypothetical protein |
| 05SSU0969 | 94 | *Streptococcus suis* 89/1591 | Type IV secretory pathway, VirB4 components |
| 05SSU0936 | 92 | *Streptococcus suis* 89/1591 | Signal recognition particle GTPase |
| 05SSU0982 | 92 | *Streptococcus suis* 89/1591 | Hypothetical protein |
| 05SSU0941 | 90 | *Streptococcus suis* 89/1591 | DNA primase (bacterial type) |
| 05SSU0974 | 89 | *Streptococcus suis* 89/1591 | Hypothetical protein |
| 05SSU0961 | 88 | *Streptococcus suis* 89/1591 | Hypothetical protein |
| 05SSU0981 | 87 | *Streptococcus suis* 89/1591 | Hypothetical protein |
| 05SSU0938 | 85 | *Streptococcus suis* 89/1591 | ATPases with chaperone activity, ATP-binding subunit |
| 05SSU0963 | 85 | *Streptococcus suis* 89/1591 | Hypothetical protein |
| 05SSU0971 | 79 | *Streptococcus suis* 89/1591 | ABC-type cobalt transport system |
| 05SSU0914 | 75 | *Streptococcus agalactiae* NEM316 | Unknown, Hypothetical protein gbs1339 |
| 05SSU0913 | 64 | *Streptococcus suis* 89/1591 | Chromosome segregation ATPases |
| 05SSU0905 | 63 | *Streptococcus agalactiae* 2603V/R | Transcriptional regulator, Cro/CI family |
| 05SSU0915 | 62 | *Streptococcus pneumoniae* R6 | Hypothetical protein spr0955 |
| 05SSU0953 | 61 | *Bacillus cereus* ATCC 10987 | DNA recombinase |
| 05SSU0910 | 48 | *Corynebacterium efficiens* YS-314 | Putative transport ATP-binding protein |
| 05SSU0943 | 47 | *Bacillus cereus* G9241 | Putative DNA-binding response regulator |
| 05SSU0907 | 46 | *Lactococcus lactis* | Nisin biosynthesis regulator NisR |
| 05SSU0952 | 44 | *Clostridium thermocellum* ATCC 27405 | Recombinase |
| 05SSU0935 | 39 | *Bacillus clausii* KSM-K16 | Hypothetical protein ABC1399 |
| 05SSU0934 | 38 | *Bacillus clausii* KSM-K16 | DNA helicase |
| 05SSU0946 | 38 | *Clostridium difficile* | Cdd4 protein |
| 05SSU0951 | 38 | *Bacillus cereus* ATCC 10987 | DNA recombinase |
| 05SSU0911 | 37 | *Leifsonia xyli* subsp. xyli str. CTCB07 | ABC transporter, NBP/MSD fusion protein |
| 05SSU0944 | 32 | *Bacillus thuringiensis* serovar konkukian str. 97-27 | Possible two-component sensor histidine kinase |
| 05SSU0949 | 29 | *Staphylococcus aureus* | Lantibiotic modifying enzyme |
| 05SSU0916 | 27 | *Streptococcus thermophilus* | Putative Abi-alpha protein |
| 05SSU0947 | 27 | *Haemophilus influenzae* R2846 | ABC-type multidrug transport system, ATPase and permease components |
| 05SSU0960 | 27 | *Streptococcus salivarius* | SalB |
| 05SSU0948 | 25 | *Lactococcus lactis* | Cytolysin B transport protein |
| 05SSU0906 | 24 | *Lactococcus lactis* | NisK |
| 05SSU0912 | 24 | *Streptomyces avermitilis* MA-4680 | Putative asparagine synthetase |
